# Supplementary material for: Ultrastructure and Physiological Characterization of Morchella Mitospores and Their Relevance in the Understanding of the Morel Life Cycle
Source: Microorganisms. 2023 Jan 30;11(2):345. doi: 10.3390/microorganisms11020345 (PMC9960803; doi:10.3390/microorganisms11020345)
Supplement: Supplementary file 1 [file microorganisms-11-00345-s001.zip › microorganisms-2145717-supplementary.pdf]

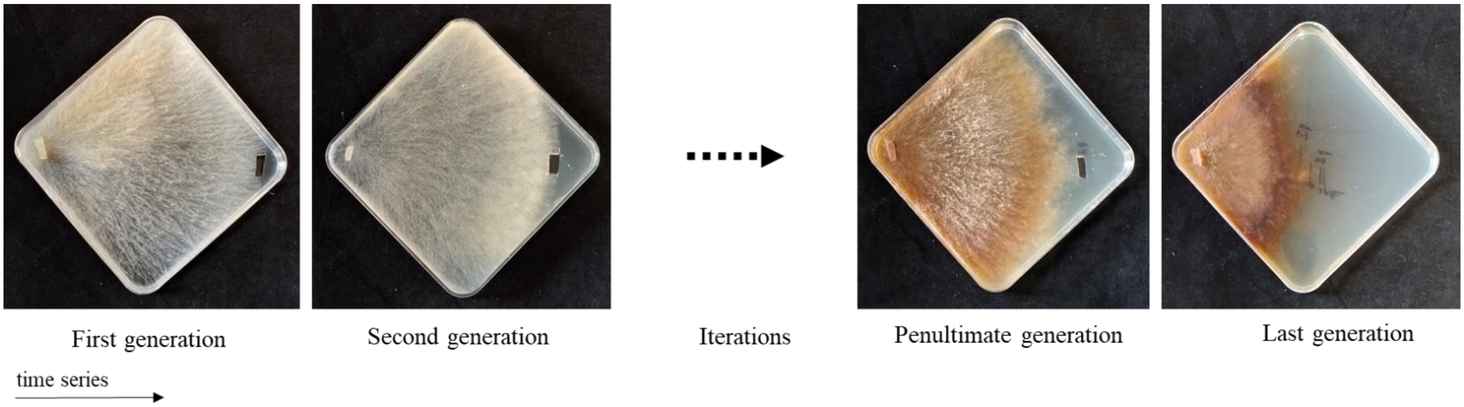

Figure S1. Method used to evaluate the life span of strains grown from morel mitospores through subculture transfers. A more detailed explanation is provided in the manuscript text.

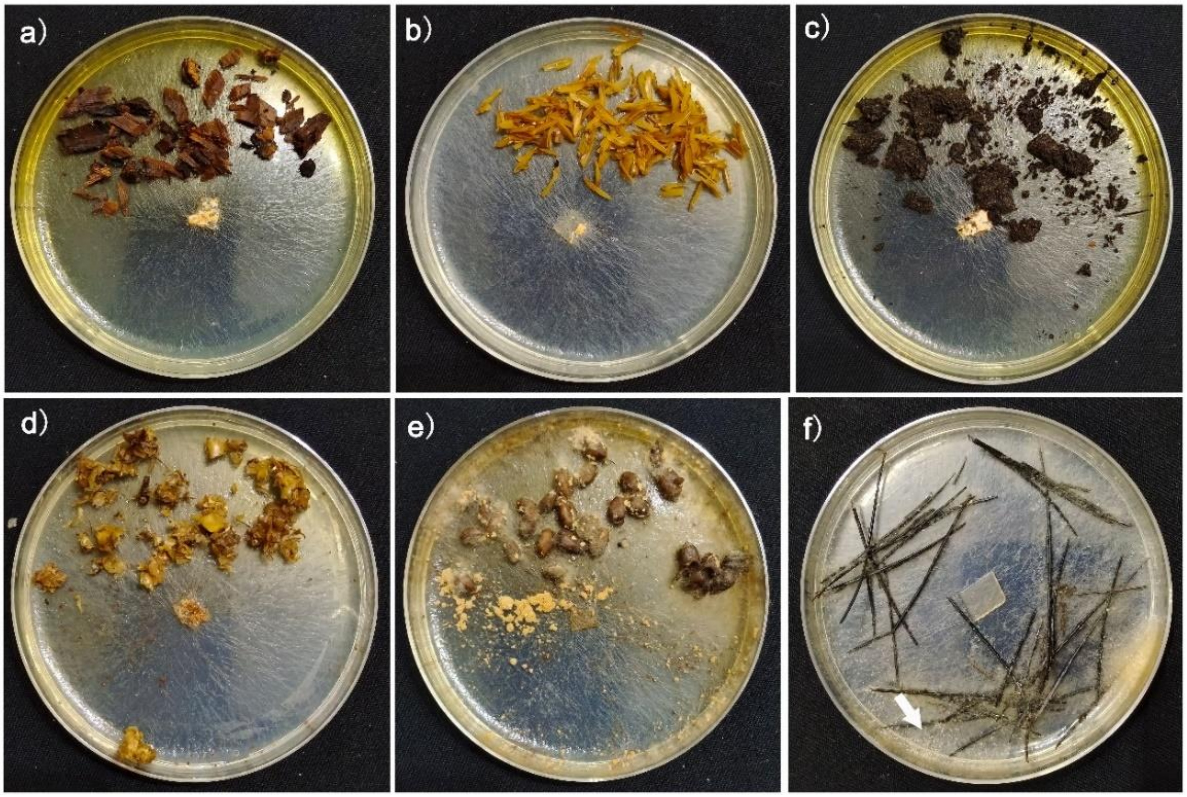

Figure S2 Mycelial growth of *Morchella sextelata* on plates with the addition of different substrates: (a) sawdust; (b) rice straw; (c) mushroom cultivation residue of *Pleurotus eryngii*; (d) corncob; (e) wheat; (f) pine needles. It is evident the lack of mitospore formation except in plates where pine needles were added (d) where occasionally some mitospore mats (arrowed) were observed.

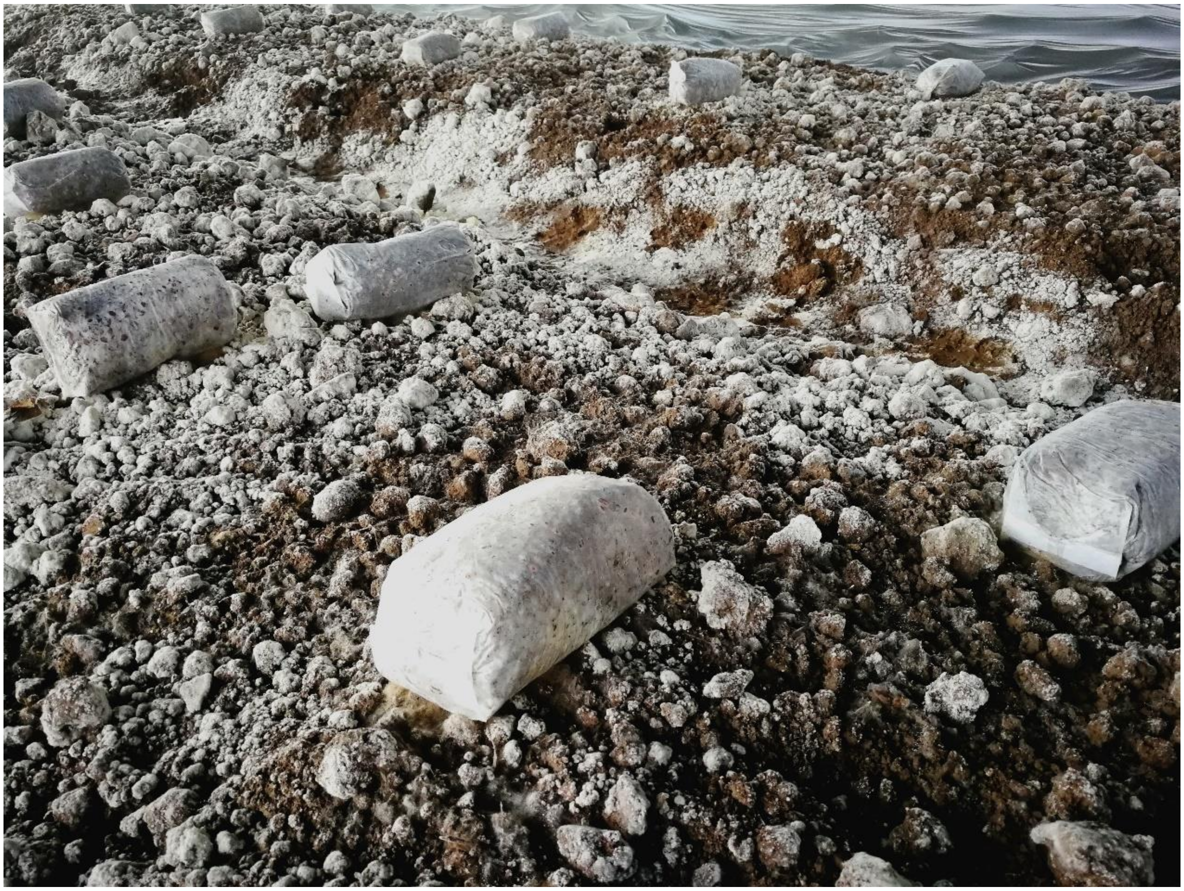

Figure S3 Abundant white and powdery mitospore mats of *Morchella sextelata* observed in field cultivation called "fungal frost". The hyphae growing on the surface are quickly transformed into white and powdery mitospore mats under the appropriate aeration and humidity conditions. The exogenous nutrition bags provide nutrition for the morel mycelia growing in colonized soil.
